# The DNA polymerases of *Drosophila melanogaster*

**DOI:** 10.1080/19336934.2019.1710076

**Published:** 2020-01-14

**Authors:** Steven J. Marygold, Helen Attrill, Elena Speretta, Kate Warner, Michele Magrane, Maria Berloco, Sue Cotterill, Mitch McVey, Yikang Rong, Masamitsu Yamaguchi

**Affiliations:** a FlyBase, Department of Physiology, Development and Neuroscience, University of Cambridge, Cambridge, UK; b UniProt, European Molecular Biology Laboratory, European Bioinformatics Institute (EMBL-EBI), Cambridgeshire, UK; c Dipartimento di Biologia, Università degli Studi di Bari “Aldo Moro”, Bari, Italy; d Department Basic Medical Sciences, St Georges University London, London, UK; e Department of Biology, Tufts University, Medford, MA, USA; f School of Life Sciences, Sun Yat-sen University, Guangzhou, China; g Department of Applied Biology and Advanced Insect Research Promotion Center, Kyoto Institute of Technology, Kyoto, Japan

**Keywords:** *Drosophila melanogaster*, DNA polymerase, DNA synthesis, DNA replication, translesion synthesis

## Abstract

DNA synthesis during replication or repair is a fundamental cellular process that is catalyzed by a set of evolutionary conserved polymerases. Despite a large body of research, the DNA polymerases of *Drosophila melanogaster* have not yet been systematically reviewed, leading to inconsistencies in their nomenclature, shortcomings in their functional (Gene Ontology, GO) annotations and an under-appreciation of the extent of their characterization. Here, we describe the complete set of DNA polymerases in *D. melanogaster*, applying nomenclature already in widespread use in other species, and improving their functional annotation. A total of 19 genes encode the proteins comprising three replicative polymerases (alpha-primase, delta, epsilon), five translesion/repair polymerases (zeta, eta, iota, Rev1, theta) and the mitochondrial polymerase (gamma). We also provide an overview of the biochemical and genetic characterization of these factors in *D. melanogaster*. This work, together with the incorporation of the improved nomenclature and GO annotation into key biological databases, including FlyBase and UniProtKB, will greatly facilitate access to information about these important proteins.

## Introduction

Multiple DNA polymerases are required for DNA synthesis in eukaryotic cells [1]. Their functions have been characterized in many different model systems and found to be highly conserved through evolution. The alpha-primase, delta and epsilon polymerases exist as multi-subunit complexes and are responsible for the bulk of nuclear DNA replication during S phase. These ‘replicative polymerases’ belong to the B family of polymerases and are characterized by relatively high processivity of 5ʹ-3ʹ DNA synthesis and high fidelity. The alpha-primase complex is responsible for synthesis of short RNA primers and initial DNA synthesis. On the lagging strand, it repeatedly synthesizes short RNA–DNA primers that are then extended by polymerase delta to generate Okazaki fragments. After initiating synthesis on the leading strand, alpha-primase is replaced by polymerase epsilon to perform the bulk of leading strand DNA elongation, though polymerase delta may be used as the leading strand polymerase in some circumstances and in certain species. Polymerase epsilon is further distinguished from delta by showing little dependence on the proliferating cell nuclear antigen (PCNA) protein for its activity. Both delta and epsilon possess a 3ʹ-5ʹ exonuclease (‘proof-reading’) activity that ensures replication fidelity. (For a recent review on replicative polymerases, see [2].)

Other specialized polymerases are required for DNA synthesis during nuclear DNA repair and/or translesion synthesis (TLS). TLS allows replication past residual DNA lesions that would otherwise block the progress of the replication machinery. On encountering a lesion, replicative DNA polymerases are switched for specific TLS polymerases (e.g. zeta, eta, iota, Rev1, theta) that then either bypass or repair lesions before the normal machinery re-associates and replication continues downstream of the damage. Owing to their specialized functions, TLS polymerases exhibit lower fidelity and processivity than replicative polymerases. TLS is mutagenic when the incorrect base is inserted opposite the DNA lesion. (For a recent review on TLS polymerases, see [3].)

TLS polymerases exhibit different preferences for types of lesions and differ in their nucleotide selection and mutagenic potential. For example, polymerase zeta (another member of the B family) is involved in the response to a large variety of DNA damage, showing high fidelity for the bypass of some lesions but low fidelity for others [4]. Polymerase eta (Y family) functions primarily to bypass cyclobutane pyrimidine dimers, which are the predominant lesions resulting from ultraviolet (UV) radiation, and can also bypass 7,8-dihydro-8-oxyguanine lesions and abasic sites [5]. In contrast to other DNA polymerases, the metazoan-specific polymerase iota (Y family) is able to use non-canonical Hoogsteen interactions for nucleotide base pairing, allowing it to incorporate nucleotides opposite various lesions in the DNA template that impair Watson-Crick interactions [6]. Rev1 (Y family) is unique in that its DNA synthesis activity is restricted to the incorporation of one or two molecules of dCTP regardless of the nature of the template nucleotide – in this sense, it is better classified as a deoxycytidyl transferase than a polymerase *per se*. Furthermore, Rev1 also has non-catalytic functions in DNA synthesis, serving as a scaffolding protein for the assembly of multiprotein TLS complexes [3]. DNA polymerase theta (belonging to the A family) is another polymerase restricted to metazoa and is unique in possessing a helicase domain associated with DNA-dependent ATPase activity [7,8]. Theta has an indispensable role in repair synthesis of double-strand breaks via an alternative non-homologous end-joining pathway and also functions in repair/bypass of interstrand crosslinks.

In contrast to the panel of polymerases functioning in the synthesis of nuclear DNA, DNA polymerase gamma (A family) is solely responsible for the replication and repair of mitochondrial DNA (mtDNA), at least in non-mammalian species (reviewed in [9]). It is highly accurate and processive, containing both polymerase and 3′-5′ exonuclease activities, as well as a 5′-deoxyribose phosphate lyase activity that functions in base excision repair. Its polymerase activity is stimulated by association with the mitochondrial single-stranded DNA binding (mtSSB). A primase for mtDNA replication has not been identified – instead, transcription is thought to provide the priming event for replication. In mammals, a second polymerase, PrimPol, is also required for repair of mtDNA damage, acting to reinitiate stalled replication forks in mitochondria [10].

While much of the work characterizing the identity and function of eukaryotic DNA polymerases has been conducted in yeast and mammalian cell culture, there is also a long and rich history of DNA polymerase research in the fruit fly, *Drosophila melanogaster*, which continues through to this day. DNA polymerase activity was first detected in fly embryos in the 1970s [11,12], which led to the purification and characterization of several replicative polymerases and their component subunits in the 1980s and 1990s. Most of the genes encoding these proteins were then cloned by individual labs in the 1990s, prior to the publication of the complete *D. melanogaster* genome sequence in 2000 [13]. Within the past two decades, all of the TLS polymerases of *D. melanogaster* have also been functionally characterized, albeit to different extents. Many fly polymerase genes have also been studied genetically, taking advantage of the relative ease and power of this approach in this model organism. These methods have been extended in recent years to create fly models of human diseases associated with polymerase dysfunction.

Despite this large body of work, the DNA polymerases of *D. melanogaster* have not yet been systematically cataloged or reviewed. This has led to inconsistent and out-dated nomenclature for this gene set and several deficiencies in their functional annotations within biological databases, which in turn has led to an under-appreciation of the extent of their characterization. Here, we seek to rectify these points. We identify all *D. melanogaster* DNA polymerases using a combination of bioinformatic and literature-based searches. In so doing, we have substantially revised and improved the Gene Ontology (GO) terms used to annotate these genes/proteins in biological databases [14]. We also propose an updated, unified nomenclature for these factors, based on that already in use in the wider field. Finally, we provide an overview of the history and key findings of the research characterizing the composition and function of DNA polymerases in flies.

## Defining the set of D. melanogaster DNA polymerases

We took a combinatorial approach to ensure we identified the full set of genes known or predicted to encode DNA polymerases in *D. melanogaster*. We searched the FlyBase [15], UniProtKB [16], QuickGO [17] and KEGG [18] databases for *D. melanogaster* entries annotated with relevant GO and/or Enzyme Commission terms. We also searched for additional literature references using FlyBase and PubMed, and identified *D. melanogaster* orthologs of the established set of human and yeast (*Saccharomyces cerevisiae*) DNA polymerases using the DIOPT and HCOP ortholog prediction tools [19,20]. The final collection of verified *D. melanogaster* polymerases/polymerase subunits is presented in Table 1.

**Table 1.** *D. melanogaster* genes encoding (subunits of) DNA polymerases

Family	Pol	Gene Symbol	Notable synonym(s)	FlyBase gene ID	UniProt protein accession	*H. sapiens* ortholog (identity)^a^	*S. cerevisiae* ortholog (identity)^a^
***Replicative polymerases***							
B	ALPHA	***PolA1***	*DNApol-ɑ180*	FBgn0259113	P26019	POLA1 (40%)	POL1 (32%)
		***PolA2***	*DNApol-ɑ73*	FBgn0005696	Q9VB62	POLA2 (30%)	POL12 (23%)
		***Prim1***	*DNApol-ɑ50*	FBgn0011762	Q24317	PRIM1 (42%)	PRI1 (36%)
		***Prim2***	*DNApol-ɑ60*	FBgn0259676	Q9VPH2	PRIM2 (33%)	PRI2 (30%)
B	DELTA	***PolD1***	*DNApol-δ*	FBgn0263600	P54358	POLD1 (58%)	POL3 (51%)
		***PolD2^b^***	*Pol31*	FBgn0027903	Q9W088	POLD2 (43%)	POL31 (30%)
		***PolD3^b^***	*Pol32, rd*	FBgn0283467	Q9Y118	POLD3 (22%)	POL32 (n/a)***^c^***
B	EPSILON	***PolE1***	*DNApol-ε255*	FBgn0264326	Q9VCN1	POLE (55%)	POL2 (39%)
		***PolE2***	*DNApol-ε58*	FBgn0035644	Q9VRQ7	POLE2 (42%)	DPB2 (25%)
		***PolE3***	*Chrac-14*	FBgn0043002	Q9V444	POLE3 (47%)	DPB4 (25%)
		***PolE4***	*Mes4*	FBgn0034726	Q9W256	POLE4 (38%)	DPB3 (28%)
***Translesion/DNA repair polymerases***							
B	ZETA	***PolZ1***	*mus205*, rev3, *DNApol-ζ*	FBgn0002891	Q9GSR1	REV3L (27%)	REV3 (25%)
		***PolZ2***	*rev7*	FBgn0037345	Q9VNE1	MAD2L2 (29%)	REV7 (18%)
		***PolD2^b^***	*Pol31*	FBgn0027903	Q9W088	POLD2 (43%)	POL31 (30%)
		***PolD3^b^***	*Pol32, rd*	FBgn0283467	Q9Y118	POLD3 (22%)	POL32 (n/a)***^c^***
Y	ETA	***PolH***	*DNApol-η* *drad30A*	FBgn0037141	Q9VNX1	POLH (29%)	RAD30 (23%)
Y	IOTA	***PolI***	*DNApol-ɩ, drad30B*	FBgn0037554	Q9VHV1	POLI (31%)	-
Y	REV1	***Rev1***	*-*	FBgn0035150	Q9W0P2	REV1 (30%)	REV1 (24%)
A	THETA	***PolQ***	*mus308, DNApol-θ*	FBgn0002905	O18475	POLQ (29%)	-
***Mitochondrial polymerase***							
A	GAMMA	***PolG1***	*tam, pol γ-α, DNApol-γ125*	FBgn0004406	Q27607	POLG (44%)	MIP1 (33%)
		***PolG2***	*pol γ-β, DNApol-ɣ35*	FBgn0004407	Q9VJV8	POLG2 (24%)	-

a. Percentage amino acid identity between the *D. melanogaster* protein and the human/yeast ortholog according to DIOPT [19]. b. PolD2 and PolD3 are part of the polymerase delta and zeta complexes. c. PolD3/Pol32 subunits are known to show extreme divergence at the primary sequence level [21], but FBgn0283467 has been identified as *D. melanogaster* PolD3/Pol32 [59].

A key part of this survey was to review and improve the GO annotations associated with the fly DNA polymerases, adding new or more specific annotations where appropriate and removing any erroneous annotations. In particular, we ensured that catalytic activity GO terms were applied only to catalytic, and not regulatory/accessory, subunits of DNA polymerase complexes. Overall, we added 140 new annotations (of which 135 are based on experimental data) and removed 10 erroneous annotations. The full set of DNA polymerase-relevant GO annotations for this set of genes/proteins following our review is provided in Supplementary Table 1.

Table 1 also shows our proposal for an updated and systematic nomenclature for the *D. melanogaster* DNA polymerase genes/proteins. This is based on the approved nomenclature used for human and vertebrate polymerases and includes the use of English rather than the traditional Greek characters, which can be difficult to interpret/process in reading the regular literature and in database representation. Previous symbols and other notable synonyms are listed in the table. (A full listing of previous and proposed nomenclature is given in Supplementary Table 2.) These revisions bring the *D. melanogaster* nomenclature into line with that used in the wider field and will make it easier for researchers from diverse backgrounds to access the fly data.

It is notable that several human/mammalian DNA polymerases lack a clear ortholog in *D. melanogaster* [19,20], namely beta (POLB), delta subunit 4 (POLD4), kappa (POLK), lambda (POLL), mu (POLM), nu (POLN), PRIMPOL and terminal deoxynucleotidyltransferase (TDT/DNTT). Conversely, *D. melanogaster* possess the iota and theta DNA polymerases that are not found in yeast.

## Research on D. melanogaster DNA polymerases

Below, we present an overview and historical perspective of the key research characterizing the composition and function of the DNA polymerases in flies, focusing on their roles in DNA synthesis (summarized in Table 2). This is not intended to be a comprehensive review of all literature concerning these factors, or how DNA polymerases function within the wider contexts of replication control and DNA repair. Readers interested in those topics are referred to excellent recent reviews [22,23].

**Table 2.** Characterization status of *D. melanogaster* DNA polymerases

	Biochemistry	Cloning	Expression	Genetics
PolA1	[28–30,33-40]	[41,42]	[24,41–45]	[47]
PolA2	[25,33–36]	[48]	[44,45,49]	[47]
Prim1	[28–36,50,51]	[51]	-	[47]
Prim2	[28–30,33-36,50]	[52]	-	[53]
PolD1	[37,56–58]	[60]	[58]	[47,58]
PolD2	[58]	n/a	[58]	[58]
PolD3	[58]	n/a	[58]	[58,59,63,64]
PolE1	[68,69]	[44,70]	[44,70]	[47,71,72]
PolE2	[69]	n/a	-	[73]
PolE3	-	n/a	-	-
PolE4	-	n/a	-	-
PolZ1	[74,75]	[76]	-	[59,76]
PolZ2	[74,75]	n/a	-	-
PolH	[80]	[80]	-	[59,81]
PolI	[80]	[80]	-	-
Rev1	[74,82]	n/a	-	[59]
PolQ	[86–88]	[83]	[87]	[88–91]
PolG1	[26,94–106]	[105]	[118]	[107–113]
PolG2	[26,94–104]	[114]	[118]	[108,117]

The references shown report biochemical data, cloning, transcript/protein expression or genetic data (i.e. phenotypic analysis using classical mutations or transgenes) relevant to the role of the gene/protein in DNA synthesis. (Cloning status is given as ‘n/a’ where cloning was not reported prior to the release of the *D. melanogaster* genome sequence.)

#### Replicative polymerases

##### Alpha (ɑ)-primase

Alpha-primase initiates synthesis on the leading strand and repeatedly synthesizes short primers on the lagging strand during nuclear DNA replication. The canonical complex comprises two primase subunits and two polymerase subunits, with each subcomplex containing one catalytic and one accessory protein (reviewed in [27]).

The *D. melanogaster* alpha-primase was first purified and characterized from embryos in a series of studies by Lehman and colleagues in the early 1980s [28–35]. This and other work [36,37] established the tetrameric subunit structure of the complex: a 182 kDa catalytic polymerase subunit (PolA1), a non-catalytic 73 kDa subunit (PolA2), and two smaller subunits of 50kDa and 60kDa (Prim1 and Prim2, respectively) associated with primase activity.

Additional research on the isolated PolA1 subunit further characterized its properties and revealed it possesses a cryptic 3ʹ-5ʹ exonuclease that functions as a proofreading activity to increase replication fidelity [38–40]. The gene encoding PolA1 was cloned in 1991–1992 [41,42]. Those and other studies [43–45] determined that levels of the protein are relatively high in unfertilized eggs and early embryos owing to maternal loading, with its subcellular location being mainly nuclear during non-mitotic phases of the cell cycle. PolA1 interacts physically and genetically with the PrSet7 methyltransferase, suggesting that this interaction and the regulation of histone monomethylation play a role in DNA replication [46]. Consistent with a key role in DNA synthesis, global RNAi-mediated knockdown of PolA1 is lethal, whereas eye-specific RNAi results in a small eye [47].

The gene encoding the 73 kDa PolA2 subunit was cloned in 1992 [48] and is expressed similarly to PolA1 [44,45,49]. Its molecular function in flies remains undefined, but is assumed to play an essential role in the initiation of DNA replication based on the characterization of orthologs [27]. Similar to PolA1, knockdown of PolA2 results in lethality or a small eye phenotype [47].

The primase subcomplex was isolated and shown capable of synthesizing primers in the absence of the other two subunits [50]. The gene encoding Prim1 was cloned in 1994 and this subunit alone was demonstrated to have primase activity [51]. The protein is detected throughout embryogenesis but is only evident in adult females (ovaries) at later development stages, suggesting maternal expression. The Prim2-encoding gene was cloned in 1999 and its expression found to be similar to Prim1 [52]. A specific function has not been ascribed to Prim2 in *D. melanogaster*, though it may stabilize Prim1 and/or act to translocate Prim1 to the nucleus based on analysis of Prim2 orthologs [27]. Knockdown of Prim1 expression causes lethality [47]. Similarly, strong *Prim2* mutations are lethal, whereas hypomorphic *Prim2* mutants show cell cycle perturbations [53].

##### Delta (δ)

DNA polymerase delta (PolD) primarily acts to extend DNA on the lagging strand, though it can also serve as the leading strand polymerase. The canonical eukaryotic complex is composed of three to four subunits, one of which possesses catalytic activity (reviewed in [54,55]).

The *D. melanogaster* PolD holoenzyme was purified from embryos and characterized to be a highly processive, PCNA-dependent DNA polymerase with a 3ʹ-5ʹ proof-reading activity [37,56,57]. It was initially identified as a heterodimer [57], but a more recent study has shown it to be a heterotrimer [58]. (Vertebrate PolD has an additional subunit, but this appears to be lacking in invertebrates.) *D. melanogaster* PolD3 (aka Pol32) facilitates nuclear localization of the PolD complex and increases PolD processivity [58,59], while PolD2 (aka Pol31) acts as a structural component, bridging the other two subunits [58].

Despite being cloned in 1995 [60], there has been relatively little genetic characterization of the *PolD1* gene – several recessive lethal mutations have been isolated [61,62] and RNAi-mediated knockdown of *PolD1* causes lethality [47]. PolD2 is similarly uncharacterized from a genetic perspective. However, genetic approaches have been used to show that PolD3 is essential for DNA replication in early embryogenesis [58,63]. Interestingly, PolD3 is also required for the repair of double-strand breaks by homologous recombination involving extensive DNA synthesis [59] and for break-induced replication during repair of single broken strands [64], though it’s unclear whether PolD functions within the polymerase delta or zeta complex in these roles (see below). Genetic interactions between PolD3 and the other two delta subunits have been reported, supporting functional associations *in vivo* [58].

##### Epsilon (ε)

Polymerase epsilon (PolE) is the major leading strand polymerase. The canonical complex is composed of one catalytic subunit and three non-catalytic subunits (reviewed in [65,66]. Recent work in mammals has shown that the two smaller PolE subunits form a stable subcomplex that acts as a histone H3-H4 chaperone to facilitate nucleosome assembly during DNA replication [67].

The *D. melanogaster* catalytic subunit (PolE1) was purified from embryos and characterized to be a highly processive DNA polymerase with a 3ʹ-5ʹ exonuclease activity [68,69]. The gene was cloned in 2000 [70] and its protein levels found to be highest in unfertilized eggs and early embryos, similar to PolA subunits [44,70]. Consistent with its molecular function, *PolE1* mutation or knock-down results in inhibition of DNA synthesis, under-sized tissues and early lethality [47,71,72]. Knock-down of *PolE1* expression also causes defective endoreplication in larval salivary glands [71,72]. Remarkably, the mitotic replication defects caused by PolE1 knock-down could be rescued by expression of the non-catalytic C-terminal domain of PolE1, whereas the endoreplication defects could not [71]. This suggests that the polymerase/exonuclease activities of PolE1 can be compensated for by other replicative polymerases (most likely PolD) during regular DNA replication in mitotic cycles, whereas these catalytic domains play a specific and critical role during endoreplication.

A 58 kDa subunit of DNApol epsilon (PolE2) was also purified and shown to directly associate with PolE1 [69]. Its specific function is unknown in flies, but it likely plays an essential structural role from studies of its orthologs [65,66]. A null mutation in the *PolE2* gene has been shown to be pupal lethal and cause defects in S phase progression in mitotic and endoreplicative cell cycles, similar to mutations in *PolE1* [73].

*D. melanogaster* PolE3 (aka Chrac-14) and PolE4 (aka Mes4) have not been studied in the context of DNA replication. Nonetheless, they are likely to be part of the *D. melanogaster* PolE holoenzyme based on studies of their orthologs and may perform similar roles to their mammalian counterparts in facilitating nucleosome assembly during replication [67].

#### Translesion/DNA repair polymerases

##### Zeta (ζ)

DNA polymerase zeta (PolZ) performs TLS in response to a variety of DNA damage. Studies in yeast and humans have shown that it exists as a heterotetramer, in which a single subunit has catalytic activity and two of the accessory subunits are shared with PolD (reviewed in [4]).

*D. melanogaster* PolZ1 was purified and characterized as a high processivity polymerase and, while lacking any 3′-5′ exonuclease function, it shows high fidelity for DNA synthesis [74]. The same study demonstrated that PolZ2 directly interacts with PolZ1 without influencing its DNA synthetic activity [74]. Interestingly, the PolZ complex may be involved in the repair of abasic sites in flies – this function is performed by DNA polymerase beta in mammals, but flies (and many other organisms) do not have a POLB ortholog [75].

The gene encoding the catalytic subunit, *PolZ1* (aka *mus205* or *rev3*), was cloned in 2001 [76]. This, and several previous genetic studies [77–79], showed that mutants were hypersensitive to alkylating agents and UV, suggesting PolZ1 is involved in lesion bypass/TLS. Further genetic characterization has demonstrated that PolZ1 also functions in the repair of double-strand breaks [59]. In contrast, there have been no genetic studies on the PolZ2 (aka Rev7) subunit to date.

While it has not been experimentally shown that the *D. melanogaster* PolZ complex includes the PolD2 and PolD3 subunits of polymerase delta, this seems likely based on studies in other species [4]. In this regard, it is possible that PolD3 acts to promote the nuclear localization of the PolZ complex in flies, as described for its function within PolD [58].

##### Eta (η)

The gene encoding *D. melanogaster* polymerase eta (PolH) was cloned in 2001 [80]. The same study demonstrated PolH has DNA polymerase activity and can bypass UV-induced lesions: *cis-syn*-cyclobutane pyrimidine dimers are bypassed error-free, whereas ([4–6])-photoproducts are bypassed in a highly error-prone manner [80]. Consistent with that study, genetic approaches have shown that PolH mutants are extremely sensitive to UV radiation [59,81]. PolH may also function in the repair synthesis of double-strand breaks [59]. Notably, the fly PolH mutants may provide *in vivo* models of a variant form of xeroderma pigmentosum, a disease characterized by increased incidence of UV-induced skin cancers caused by mutations in human PolH [81].

##### Iota (ι)

Similar to PolH, the gene encoding *D. melanogaster* polymerase iota (PolI) was cloned in 2001 and the purified protein shown to have DNA polymerase activity [80]. It too can bypass *cis-syn*-cyclobutane pyrimidine dimers UV photoproducts in an error-free manner, though it is not able to bypass ([4–6])-photoproducts at all [80]. PolI function has not been studied using genetic approaches to date, and it remains an open question whether it plays roles outside of being a ‘backup’ to PolH.

##### Rev1

A scaffolding/co-ordinating function for *D. melanogaster* Rev1 is supported by studies demonstrating physical interactions between it and other TLS polymerases including PolH, PolI, PolZ1 and PolZ2 [74,82]. Genetic investigations lend further support to this view, showing that loss of Rev1 results in high sensitivity to ionizing radiation and affects the degree of homologous recombination repair synthesis [59]. One idea arising from these studies is that Rev1 coordinates the initial recruitment of other translesion polymerases and, in so doing, prevents replicative polymerases from acting during early repair synthesis [59]. There are no biochemical data supporting a deoxycytidyl transferase/DNA polymerase activity of Rev1 in flies, though this is assumed to be present from characterization of its orthologs.

##### Theta (θ)

PolQ is the best studied TLS polymerase in *D. melanogaster*. The gene encoding PolQ was cloned in 1996 [83] and shown to correspond to the well-studied *mutagen-sensitive 308* (*mus308*) locus, mutations in which conferred strong sensitivity to DNA cross-linking agents [84,85]. The purified PolQ protein was demonstrated to have both polymerase and, uniquely amongst DNA polymerases, DNA-dependent ATPase activity [86–88]. It is expressed throughout all stages of development [87].

The ATPase activity of PolQ is associated with a N-terminal helicase-like domain, which is separated from the C-terminal polymerase domain by an unstructured central region [83]. Both domains are required for resistance to cross-linking agents *in vivo* and purified PolQ can bypass structures representative of unhooked crosslinks *in vitro* [88]. These data suggest that the helicase activity may stimulate DNA unwinding and strand displacement to facilitate synthesis by the polymerase domain.

A series of genetic studies by the McVey laboratory have delineated a second, crucial function for PolQ in repair of double-strand breaks via a process termed microhomology-mediated end-joining (MMEJ) [88–91]. MMEJ occurs when short, complementary DNA sequences located at broken DNA ends anneal and serve as primers for fill-in synthesis. While only the polymerase activity of PolQ is required for MMEJ, loss of the helicase domain affects the spectrum of repair junctions that are recovered [88]. PolQ is also important for repair of double-strand breaks arising from replication fork collapse during endoreplication in eggshell-producing follicle cells [91].

#### Mitochondrial polymerase

##### Gamma (γ)

DNA polymerase gamma (PolG) is responsible for the replication and repair of mtDNA (reviewed in [9]. The subunit composition of the holoenzyme varies across species, ranging from a single catalytic subunit in yeast and nematodes to a heterodimer (one accessory subunit) in insects, and a heterotrimer (including a dimeric accessory subunit) in vertebrates [92].

PolG is the best characterized *D. melanogaster* polymerase, having been comprehensively analyzed by the Kaguni laboratory over several years [93]. The complex was first purified from embryos in 1986 and found to comprise two polypeptides of 125 and 35 kDa [94]. Several subsequent studies defined the holoenzyme as a high fidelity polymerase associated with a 3ʹ-5ʹ exonuclease (proof-reading) activity, which requires the mitochondrial single stranded binding protein (mtSSB) to stimulate its processivity [95–104].

The gene encoding the large subunit, PolG1, was cloned in 1996 [105]. This and other studies established that PolG1 harbors both the polymerase and exonuclease activities, as well as a mitochondrial targeting sequence [102,105,106]. Mutations in this gene were first identified via a screen for altered larval responses to light (resulting in the gene being named ‘*tamas*’ – Sanskrit for ‘dark inertia’) – indeed, these were the first reported mutations in any PolG gene [107]. Mutants die during late larval stages, exhibiting impaired mtDNA replication and decreased mtDNA content [107–109]. Ubiquitous RNAi-mediated knock-down of PolG1 depletes mtDNA, decreases mitochondrial respiratory activity and results in lethality, while neuronal-specific knock-down causes progressive behavioral deficits [110,111]. Other studies have utilized sophisticated genetic techniques to engineer flies to express either polymerase- or exonuclease-deficient PolG1 – the former resulted in mtDNA depletion, whereas the latter led to accumulation of mutations/deletions of mitochondrial DNA [112,113].

The gene encoding the smaller accessory subunit, PolG2, was cloned in 1997 [114]. It is located in the genome within 9 kilobases of the *PolG1* gene and the encoded protein contains a mitochondrial targeting sequence [102,114]. Biochemical analyses have demonstrated a critical role for PolG2 in the catalytic efficiency of the PolG1 subunit [102], while structural modeling suggests it is also involved in primer recognition and processive DNA elongation [115]. These findings are consistent with work on the mammalian ortholog [116] and define the PolG2 subunit as a processivity factor for the PolG1 polymerase. Null mutations in *PolG2* have been generated and result in lethality during pupal stages, with mutant cells exhibiting loss of mtDNA and reduced proliferation [108,117].

Despite their proximity in the genome, the *PolG1* and *PolG2* genes exhibit differential expression: *PolG1* is expressed highly in very early embryos but is then largely undetectable until adult stages, whereas *PolG2* expression increases during embryonic stages, remains present at lower levels during larval and pupal development, and is then expressed highly again in adults [118]. One reason for this difference is that transcription of *PolG2*, but not *PolG1*, is in part governed by a DNA replication-related element (DRE) in its promoter [118]. Interestingly, DREs have also been identified in the promoters of the *PolA1, PolA2, PolE4* and *PCNA* genes [41,49,119,120] and thus may be widely used to control the expression of DNA replication-related factors.

Mutations in human *POLG* and *POLG2* are associated with several diseases including mtDNA depletion syndromes, such as Alpers syndrome, and mtDNA deletion disorders, such as progressive external ophthalmoplegia [121]. Significantly, several studies in the last decade have manipulated the orthologous *D. melanogaster* genes to provide important insights into the etiology of, and possible therapies for, these diseases [108–113,122]. For example, two studies generated flies expressing exonuclease- or polymerase-deficient versions of PolG1 in order to investigate how defects in these two different activities of the enzyme may contribute to pathophysiology [112,113]. Two other investigations studied the effects of mutations or knock-down of *D. melanogaster PolG1* or *PolG2* specifically in the nervous system to probe how mitochondrial dysfunction may lead to neuropathies and neurodegenerative disorders in humans [108,110]. As a final example, Siibak et al. engineered the endogenous *PolG1* gene in flies to have mutations equivalent to those found in patients to probe their molecular and physiological consequences, finding that both mutations cause mtDNA depletion [109].

## Perspective

The DNA polymerases of *D. melanogaster* have been researched for almost 50 years. In this brief report, we have gathered together the fruits of these investigations and presented the full set of fly DNA polymerases alongside the key references characterizing them. In so doing, we have proposed a revised nomenclature that is in line with wider usage in the field and greatly improved the quality and coverage of the functional (GO) annotations associated with these factors. These improvements in annotation and their integration into core biological databases, including FlyBase (www.flybase.org) and UniProtKB (www.uniprot.org), will greatly enhance access to, and the impact of, *D. melanogaster* DNA polymerase research. Furthermore, all UniProtKB records for these proteins have been manually annotated/reviewed and a dedicated ‘Gene Group’ page for the DNA polymerases has been added to FlyBase (http://flybase.org/reports/FBgg0001200).

Much of what we know about eukaryotic polymerases has been conducted in yeast and mammalian cell culture. Flies provide a way to investigate polymerase function in the context of an intact multi-cellular organism with unrivaled possibilities for sophisticated genetic analyses to inform biological function. Indeed, the genetic approach, often combined with biochemical assays, has been key to the several landmark discoveries about DNA polymerases that were made through research using *D. melanogaster*. These include the first characterization of mutations in PolG subunits [107,117], which ultimately led to a genetic dissection of the roles of the exonuclease and polymerase domains of PolG1 [112,113]; the function of PolQ within the MMEJ repair pathway [88–90]; and the role of PolD3 in facilitating the nuclear import of PolD1 [58]. Several of the mechanisms elucidated by these studies provided precedent for other organisms, particularly so in the case of PolQ. For example, studies of PolQ in *C. elegans*, mice, and human cells have clearly shown that both its domains are important for alternative end joining/MMEJ processes [123–125], as originally described in flies. The role of *D. melanogaster* PolQ in the repair of double-strand breaks arising from replication fork collapse [91] has also been found to be conserved [126–128]. Early studies on the fly PolQ identified its role in interstrand crosslink repair – a similar role for mammalian PolQ has been debated [7], but a recent study has demonstrated that human POLQ is required for repair of mitomycin C-induced damage, which introduces interstrand crosslinks [127]. These examples emphasize the relevance of work on *D. melanogaster* DNA polymerases to other eukaryotic animals, including humans.

Nonetheless, it’s equally clear that flies must do a few things differently, at least compared to the well-studied yeast and mammalian polymerase systems. For instance, flies have two additional polymerases (iota and theta) compared to yeast, but lack several of the TLS polymerases present in mammals (Table 1). Presumably, the ‘missing’ polymerases are either not required for DNA synthesis in yeast/fly cells or their functions are performed by one or more of the polymerases that are present [75]. The subunit composition of the delta and gamma polymerases also differs between these three species (Table 1), leading to functional differences at least in the latter case [92]. As a final example, several fly cell types exhibit endoreplication, whereby cells undergo multiple S phases without entering mitosis or cytokinesis, resulting in giant cells with a polyploid nucleus [129]. This variant cell cycle is common in other arthropods, nematodes and plants, but is rarely found in mammals and is absent from yeast. While regulation of endoreplication occurs primarily at the level of core components of the cell cycle machinery, it’s apparent that some fly DNA polymerases, including PolE and PolQ have also developed endocycle-specific regulatory mechanisms [71–73,91].

We anticipate that future research using *D. melanogaster* will continue to provide pioneering insights into DNA polymerase biology that are applicable across a wide range of species. Pursuing and developing additional fly models of the several diseases associated with lesions in human DNA polymerases [121,130] is likely to be a particularly useful research direction. We hope the work presented in this review will facilitate these new studies and aid the integration of the ensuing knowledge.

## Supplementary Material

Supplemental Material
