# Supplementary material for: The DNA polymerases of Drosophila melanogaster
Source: Fly (Austin). 2020 Jan 14;14(1-4):49–61. doi: 10.1080/19336934.2019.1710076 (PMC7714529; doi:10.1080/19336934.2019.1710076)
Supplement: Supplemental Material [file KFLY_A_1710076_SM2276.docx]

**Supplementary Table 1. DNA polymerase-relevant Gene Ontology (GO) annotations for the DNA polymerases of *D. melanogaster***

Aspect abbreviations: BP = biological process; MF = molecular function; CC = cellular component. ‘Relevant annotations’ are defined as manual (i.e. not computational) annotations using BP terms mapping under ‘DNA biosynthetic process’ (GO:007189) and/or ‘DNA replication’ (GO:0006260); MF terms ‘DNA-directed DNA polymerase activity’ (GO:0003887), ‘DNA primase activity’ (GO:0003896), ‘3'-5'-exodeoxyribonuclease activity’ (GO:0008296), DNA polymerase processivity factor activity (GO:0030337) or ‘deoxycytidyl transferase activity’ (GO:0017125) (for Rev1); and CC terms mapping under ‘DNA polymerase complex’ (GO:0042575). Evidence abbreviations: IDA = inferred from direct assay; IPI = inferred from physical interaction; IMP = inferred from mutant phenotype; ISS = inferred from sequence similarity. IDA, IPI and IMP indicate there is experimental evidence for the given GO term (shown in blue); ISS means that the GO annotation is based on sequence similarity to another gene, and is only shown here where experimental data are lacking. Data were obtained from the Gene Ontology Annotation database (1), accessed 29th November 2019.

| **Symbol** | **GO annotations** | | | |
| --- | --- | --- | --- | --- |
|  | **Aspect** | **GO term (ID)** | **Evidence** | **Reference(s)** |
| PolA1 | BP | DNA-dependent DNA replication (GO:0006261)  DNA replication proofreading (GO:0045004) | IDA  IDA | (2-8)  (6,9) |
|  | MF | DNA-directed DNA polymerase activity (GO:0003887)  3'-5'-exodeoxyribonuclease activity (GO:0008296) | IDA  IDA | (2-5,7,8,10)  (6,9) |
|  | CC | alpha DNA polymerase:primase complex (GO:0005658) | IDA | (2-5,7,8) |
| PolA2 | BP | DNA-dependent DNA replication (GO:0006261) | IDA | (4,7) |
|  | MF |  |  |  |
|  | CC | alpha DNA polymerase:primase complex (GO:0005658) | IDA | (3,4,7) |
| Prim1 | BP | DNA-dependent DNA replication (GO:0006261)  DNA replication, synthesis of RNA primer (GO:0006269) | IDA  IDA | (2,4,7)  (3,7,11,12) |
|  | MF | DNA primase activity (GO:0003896) | IDA | (12) |
|  | CC | alpha DNA polymerase:primase complex (GO:0005658) | IDA | (2-4,7,11,12) |
| Prim2 | BP | DNA-dependent DNA replication (GO:0006261)  DNA replication, synthesis of RNA primer (GO:0006269) | IDA  IDA | (2,4,7)  (3,7,11) |
|  | MF |  |  |  |
|  | CC | alpha DNA polymerase:primase complex (GO:0005658) | IDA | (2-4,7,11) |
| PolD1 | BP | DNA-dependent DNA replication (GO:0006261)  DNA replication proofreading (GO:0045004) | IDA  IDA | (8,13,14)  (14) |
|  | MF | DNA-directed DNA polymerase activity (GO:0003887)  3'-5'-exodeoxyribonuclease activity (GO:0008296) | IDA  IDA | (8,13,14)  (8,13,14) |
|  | CC | delta DNA polymerase complex (GO:0043625) | IDA | (8,15) |
| PolD2 | BP | mitotic DNA-dependent DNA replication (GO:1990506) | IMP | (15) |
|  | MF |  |  |  |
|  | CC | delta DNA polymerase complex (GO:0043625)  zeta DNA polymerase complex (GO:0016035) | IDA  ISS | (15)  n/a |
| PolD3 | BP | DNA synthesis involved in double-strand break repair via homologous recombination (GO:0043150)  mitotic DNA replication (GO:1902969)  positive regulation of DNA-directed DNA polymerase activity (GO:1900264) | IMP  IMP  IMP | (16)  (17)  (16) |
|  | MF | DNA polymerase processivity factor activity (GO:0030337) | IMP | (16) |
|  | CC | delta DNA polymerase complex (GO:0043625)  zeta DNA polymerase complex (GO:0016035) | IDA  ISS | (15)  n/a |
| PolE1 | BP | DNA-dependent DNA replication (GO:0006261)  DNA replication proofreading (GO:0045004)  DNA synthesis involved in DNA replication (GO:0090592) | IDA  IDA  IDA | (18)  (19)  (19) |
|  | MF | DNA-directed DNA polymerase activity (GO:0003887)  3'-5'-exodeoxyribonuclease activity (GO:0008296) | IDA  IDA | (18,19)  (18,19) |
|  | CC | epsilon DNA polymerase complex (GO:0008622) | IPI | (19) |
| PolE2 | BP | DNA replication proofreading (GO:0045004)  mitotic DNA replication (GO:1902969) | IDA  IMP | (19)  (20) |
|  | MF |  |  |  |
|  | CC | epsilon DNA polymerase complex (GO:0008622) | IPI | (19) |
| PolE3 | BP | DNA-dependent DNA replication (GO:0006261) | ISS | n/a |
|  | MF |  |  |  |
|  | CC | epsilon DNA polymerase complex (GO:0008622) | ISS | n/a |
| PolE4 | BP | DNA-dependent DNA replication (GO:0006261) | ISS | n/a |
|  | MF |  |  |  |
|  | CC | epsilon DNA polymerase complex (GO:0008622) | ISS | n/a |
| PolZ1 | BP | DNA synthesis involved in double-strand break repair via homologous recombination (GO:0043150)  DNA biosynthetic process (GO:0071897)  DNA synthesis involved in DNA repair (GO:0000731) | IMP  IDA  IDA | (16)  (21)  (22) |
|  | MF | DNA-directed DNA polymerase activity (GO:0003887) | IDA | (21) |
|  | CC | zeta DNA polymerase complex (GO:0016035) | IPI | (21) |
| PolZ2 | BP |  |  |  |
|  | MF |  |  |  |
|  | CC | zeta DNA polymerase complex (GO:0016035) | IPI | (21) |
| PolH | BP | translesion synthesis (GO:0019985) | IDA | (23) |
|  | MF | DNA-directed DNA polymerase activity (GO:0003887) | IDA, IMP | (23) |
|  | CC | n/a |  |  |
| PolI | BP | translesion synthesis (GO:0019985) | IDA | (23) |
|  | MF | DNA-directed DNA polymerase activity (GO:0003887) | IDA, IMP | (23) |
|  | CC | n/a |  |  |
| Rev1 | BP | DNA synthesis involved in double-strand break repair via homologous recombination (GO:0043150)  translesion synthesis (GO:0019985) | IMP  ISS | (16)  n/a |
|  | MF | deoxycytidyl transferase activity (GO:0017125) | ISS | n/a |
|  | CC | n/a |  |  |
| PolQ | BP | DNA synthesis involved in DNA repair (GO:0000731)  DNA synthesis involved in DNA repair (GO:0000731)  DNA biosynthetic process (GO:0071897) | IDA  IMP  IDA | (24)  (25)  (26) |
|  | MF | DNA-directed DNA polymerase activity (GO:0003887) | IDA | (24,26,27) |
|  | CC | n/a |  |  |
| PolG1 | BP | DNA-dependent DNA replication (GO:0006261)  mitochondrial DNA replication (GO:0006264)  DNA replication proofreading (GO:0045004)  DNA replication proofreading (GO:0045004) | IDA  IMP  IDA  IMP | (28-41)  (42-44)  (33,35)  (43) |
|  | MF | DNA-directed DNA polymerase activity (GO:0003887)  DNA-directed DNA polymerase activity (GO:0003887)  3'-5'-exodeoxyribonuclease activity (GO:0008296)  3'-5'-exodeoxyribonuclease activity (GO:0008296) | IDA  IMP  IDA  IMP | (28,33,35,37,38,40,41)  (43)  (33,35,37,40)  (43) |
|  | CC | gamma DNA polymerase complex (GO:0005760) | IDA | (28,33,37,38,40,41) |
| PolG2 | BP | DNA-dependent DNA replication (GO:0006261)  DNA-dependent DNA replication (GO:0006261)  mitochondrial DNA replication (GO:0006264)  positive regulation of DNA-directed DNA polymerase activity (GO:1900264) | IDA  IPI  IMP  ISS | (28-34,36-41)  (45)  (42)  (46) |
|  | MF | DNA polymerase processivity factor activity (GO:0030337) | ISS | (46) |
|  | CC | gamma DNA polymerase complex (GO:0005760)  gamma DNA polymerase complex (GO:0005760) | IPI  IDA | (45)  (28,33,37,38,40,41) |

**Supplementary Table 2. Proposed and previous FlyBase symbols/names for the DNA polymerases of *D. melanogaster***

| **Proposed symbol** | **Proposed name** | **Previous symbol** | **Previous name** | **FBgn ID** | **CG number** |
| --- | --- | --- | --- | --- | --- |
| PolA1 | DNA polymerase alpha subunit 1 | DNApol-ɑ180 | DNA polymerase ɑ 180kD | FBgn0259113 | CG6349 |
| PolA2 | DNA polymerase alpha subunit 2 | DNApol-ɑ73 | DNA polymerase ɑ 73kD | FBgn0005696 | CG5923 |
| Prim1 | DNA primase subunit 1 | DNApol-ɑ50 | DNA polymerase ɑ 50kD | FBgn0011762 | CG7108 |
| Prim2 | DNA primase subunit 2 | DNApol-ɑ60 | DNA polymerase ɑ 60kD | FBgn0259676 | CG5553 |
| PolD1 | DNA polymerase delta subunit 1 | DNApol-δ | DNA-polymerase-delta | FBgn0263600 | CG5949 |
| PolD2 | DNA polymerase delta subunit 2 | Pol31 | Pol31 polymerase delta subunit | FBgn0027903 | CG12018 |
| PolD3 | DNA polymerase delta subunit 3 | Pol32 | Pol32 polymerase delta subunit | FBgn0283467 | CG3975 |
| PolE1 | DNA polymerase epsilon subunit 1 | DNApol-ε255 | DNA polymerase ε 255kD subunit | FBgn0264326 | CG6768 |
| PolE2 | DNA polymerase epsilon subunit 2 | DNApol-ε58 | DNA polymerase ε 58kD subunit | FBgn0035644 | CG10489 |
| PolE3 | DNA polymerase epsilon subunit 3 | Chrac-14 | Chromatin accessibility complex 14kD protein | FBgn0043002 | CG13399 |
| PolE4 | DNA polymerase epsilon subunit 4 | Mes4 | Mesoderm-expressed 4 | FBgn0034726 | CG11301 |
| PolZ1 | DNA polymerase zeta subunit 1 | mus205 | mutagen-sensitive 205 | FBgn0002891 | CG1925 |
| PolZ2 | DNA polymerase zeta subunit 2 | rev7 | rev7 | FBgn0037345 | CG2948 |
| PolH | DNA polymerase eta | DNApol-η | DNApol-η | FBgn0037141 | CG7143 |
| PolI | DNA polymerase iota | DNApol-ɩ | DNApol-ɩ | FBgn0037554 | CG7602 |
| Rev1 | DNA polymerase Rev1 | Rev1 | Rev1 | FBgn0035150 | CG12189 |
| PolQ | DNA polymerase theta | mus308 | mutagen-sensitive 308 | FBgn0002905 | CG6019 |
| PolG1 | DNA polymerase gamma subunit 1 | tam | tamas | FBgn0004406 | CG8987 |
| PolG2 | DNA polymerase gamma subunit 2 | DNApol-ɣ35 | DNA polymerase gamma 35kD | FBgn0004407 | CG33650 |

**Supplemental References**

1. Huntley, R.P., Sawford, T., Mutowo-Meullenet, P., Shypitsyna, A., Bonilla, C., Martin, M.J. and O'Donovan, C. (2015) The GOA database: gene Ontology annotation updates for 2015. *Nucleic Acids Res*, **43**, D1057-1063.

2. Villani, G., Sauer, B. and Lehman, I.R. (1980) DNA polymerase alpha from Drosophila melanogaster embryos. Subunit structure. *J Biol Chem*, **255**, 9479-9483.

3. Kaguni, L.S., Rossignol, J.M., Conaway, R.C., Banks, G.R. and Lehman, I.R. (1983) Association of DNA primase with the beta/gamma subunits of DNA polymerase alpha from Drosophila melanogaster embryos. *J Biol Chem*, **258**, 9037-9039.

4. Kaguni, L.S., Rossignol, J.M., Conaway, R.C. and Lehman, I.R. (1983) Isolation of an intact DNA polymerase-primase from embryos of Drosophila melanogaster. *Proc Natl Acad Sci U S A*, **80**, 2221-2225.

5. Cotterill, S., Chui, G. and Lehman, I.R. (1987) DNA polymerase-primase from embryos of Drosophila melanogaster. The DNA polymerase subunit. *J Biol Chem*, **262**, 16100-16104.

6. Reyland, M.E., Lehman, I.R. and Loeb, L.A. (1988) Specificity of proofreading by the 3'----5' exonuclease of the DNA polymerase-primase of Drosophila melanogaster. *J Biol Chem*, **263**, 6518-6524.

7. Kuroda, K., Kagiyama-Takahashi, R. and Shinomiya, T. (1990) Immunoaffinity purification and properties of Drosophila melanogaster DNA polymerase alpha-primase complex. *J Biochem*, **108**, 926-933.

8. Peck, V.M., Gerner, E.W. and Cress, A.E. (1992) Delta-type DNA polymerase characterized from Drosophila melanogaster embryos. *Nucleic Acids Res*, **20**, 5779-5784.

9. Cotterill, S.M., Reyland, M.E., Loeb, L.A. and Lehman, I.R. (1987) A cryptic proofreading 3'----5' exonuclease associated with the polymerase subunit of the DNA polymerase-primase from Drosophila melanogaster. *Proc Natl Acad Sci U S A*, **84**, 5635-5639.

10. Kaguni, L.S., DiFrancesco, R.A. and Lehman, I.R. (1984) The DNA polymerase-primase from drosophila melanogaster embryos. Rate and fidelity of polymerization on single-stranded DNA templates. *J Biol Chem*, **259**, 9314-9319.

11. Cotterill, S., Chui, G. and Lehman, I.R. (1987) DNA polymerase-primase from embryos of Drosophila melanogaster. DNA primase subunits. *J Biol Chem*, **262**, 16105-16108.

12. Bakkenist, C.J. and Cotterill, S. (1994) The 50-kDa primase subunit of Drosophila melanogaster DNA polymerase alpha. Molecular characterization of the gene and functional analysis of the overexpressed protein. *J Biol Chem*, **269**, 26759-26766.

13. Chiang, C.S., Mitsis, P.G. and Lehman, I.R. (1993) DNA polymerase delta from embryos of Drosophila melanogaster. *Proc Natl Acad Sci U S A*, **90**, 9105-9109.

14. Aoyagi, N., Matsuoka, S., Furunobu, A., Matsukage, A. and Sakaguchi, K. (1994) Drosophila DNA polymerase delta. Purification and characterization. *J Biol Chem*, **269**, 6045-6050.

15. Ji, J., Tang, X., Hu, W., Maggert, K.A. and Rong, Y.S. (2019) The processivity factor Pol32 mediates nuclear localization of DNA polymerase delta and prevents chromosomal fragile site formation in Drosophila development. *PLoS Genet*, **15**, e1008169.

16. Kane, D.P., Shusterman, M., Rong, Y. and McVey, M. (2012) Competition between replicative and translesion polymerases during homologous recombination repair in Drosophila. *PLoS Genet*, **8**, e1002659.

17. Tritto, P., Palumbo, V., Micale, L., Marzulli, M., Bozzetti, M.P., Specchia, V., Palumbo, G., Pimpinelli, S. and Berloco, M. (2015) Loss of Pol32 in Drosophila melanogaster causes chromosome instability and suppresses variegation. *PLoS One*, **10**, e0120859.

18. Aoyagi, N., Oshige, M., Hirose, F., Kuroda, K., Matsukage, A. and Sakaguchi, K. (1997) DNA polymerase epsilon from Drosophila melanogaster. *Biochem Biophys Res Commun*, **230**, 297-301.

19. Oshige, M., Takeuchi, R., Ruike, T., Kuroda, K. and Sakaguchi, K. (2004) Subunit protein-affinity isolation of Drosophila DNA polymerase epsilon catalytic subunit. *Protein Expr Purif*, **35**, 248-256.

20. Sahashi, R., Matsuda, R., Suyari, O., Kawai, M., Yoshida, H., Cotterill, S. and Yamaguchi, M. (2013) Functional analysis of Drosophila DNA polymerase epsilon p58 subunit. *Am J Cancer Res*, **3**, 478-489.

21. Takeuchi, R., Oshige, M., Uchida, M., Ishikawa, G., Takata, K., Shimanouchi, K., Kanai, Y., Ruike, T., Morioka, H. and Sakaguchi, K. (2004) Purification of Drosophila DNA polymerase zeta by REV1 protein-affinity chromatography. *Biochem J*, **382**, 535-543.

22. Takeuchi, R., Ruike, T., Nakamura, R., Shimanouchi, K., Kanai, Y., Abe, Y., Ihara, A. and Sakaguchi, K. (2006) Drosophila DNA polymerase zeta interacts with recombination repair protein 1, the Drosophila homologue of human abasic endonuclease 1. *J Biol Chem*, **281**, 11577-11585.

23. Ishikawa, T., Uematsu, N., Mizukoshi, T., Iwai, S., Iwasaki, H., Masutani, C., Hanaoka, F., Ueda, R., Ohmori, H. and Todo, T. (2001) Mutagenic and nonmutagenic bypass of DNA lesions by Drosophila DNA polymerases dpoleta and dpoliota. *J Biol Chem.*, **276**, 15155-15163.

24. Beagan, K., Armstrong, R.L., Witsell, A., Roy, U., Renedo, N., Baker, A.E., Scharer, O.D. and McVey, M. (2017) Drosophila DNA polymerase theta utilizes both helicase-like and polymerase domains during microhomology-mediated end joining and interstrand crosslink repair. *PLoS Genet*, **13**, e1006813.

25. Chan, S.H., Yu, A.M. and McVey, M. (2010) Dual roles for DNA polymerase theta in alternative end-joining repair of double-strand breaks in Drosophila. *PLoS Genet*, **6**, e1001005.

26. Oshige, M., Aoyagi, N., Harris, P.V., Burtis, K.C. and Sakaguchi, K. (1999) A new DNA polymerase species from Drosophila melanogaster: a probable mus308 gene product. *Mutat Res*, **433**, 183-192.

27. Pang, M., McConnell, M. and Fisher, P.A. (2005) The Drosophila mus 308 gene product, implicated in tolerance of DNA interstrand crosslinks, is a nuclear protein found in both ovaries and embryos. *DNA Repair (Amst)*, **4**, 971-982.

28. Wernette, C.M. and Kaguni, L.S. (1986) A mitochondrial DNA polymerase from embryos of Drosophila melanogaster. Purification, subunit structure, and partial characterization. *J Biol Chem*, **261**, 14764-14770.

29. Wernette, C.M., Conway, M.C. and Kaguni, L.S. (1988) Mitochondrial DNA polymerase from Drosophila melanogaster embryos: kinetics, processivity, and fidelity of DNA polymerization. *Biochemistry*, **27**, 6046-6054.

30. Kaguni, L.S. and Olson, M.W. (1989) Mismatch-specific 3'----5' exonuclease associated with the mitochondrial DNA polymerase from Drosophila embryos. *Proc Natl Acad Sci U S A*, **86**, 6469-6473.

31. Olson, M.W. and Kaguni, L.S. (1992) 3'-->5' exonuclease in Drosophila mitochondrial DNA polymerase. Substrate specificity and functional coordination of nucleotide polymerization and mispair hydrolysis. *J Biol Chem*, **267**, 23136-23142.

32. Williams, A.J., Wernette, C.M. and Kaguni, L.S. (1993) Processivity of mitochondrial DNA polymerase from Drosophila embryos. Effects of reaction conditions and enzyme purity. *J Biol Chem*, **268**, 24855-24862.

33. Olson, M.W., Wang, Y., Elder, R.H. and Kaguni, L.S. (1995) Subunit structure of mitochondrial DNA polymerase from Drosophila embryos. Physical and immunological studies. *J Biol Chem*, **270**, 28932-28937.

34. Williams, A.J. and Kaguni, L.S. (1995) Stimulation of Drosophila mitochondrial DNA polymerase by single-stranded DNA-binding protein. *J Biol Chem*, **270**, 860-865.

35. Lewis, D.L., Farr, C.L., Wang, Y., Lagina, A.T., 3rd and Kaguni, L.S. (1996) Catalytic subunit of mitochondrial DNA polymerase from Drosophila embryos. Cloning, bacterial overexpression, and biochemical characterization. *J Biol Chem*, **271**, 23389-23394.

36. Farr, C.L., Wang, Y. and Kaguni, L.S. (1999) Functional interactions of mitochondrial DNA polymerase and single-stranded DNA-binding protein. Template-primer DNA binding and initiation and elongation of DNA strand synthesis. *J Biol Chem*, **274**, 14779-14785.

37. Wang, Y. and Kaguni, L.S. (1999) Baculovirus expression reconstitutes Drosophila mitochondrial DNA polymerase. *J Biol Chem*, **274**, 28972-28977.

38. Fan, L. and Kaguni, L.S. (2001) Multiple regions of subunit interaction in Drosophila mitochondrial DNA polymerase: three functional domains in the accessory subunit. *Biochemistry*, **40**, 4780-4791.

39. Farr, C.L., Matsushima, Y., Lagina, A.T., 3rd, Luo, N. and Kaguni, L.S. (2004) Physiological and biochemical defects in functional interactions of mitochondrial DNA polymerase and DNA-binding mutants of single-stranded DNA-binding protein. *J Biol Chem*, **279**, 17047-17053.

40. Luo, N. and Kaguni, L.S. (2005) Mutations in the spacer region of Drosophila mitochondrial DNA polymerase affect DNA binding, processivity, and the balance between Pol and Exo function. *J Biol Chem*, **280**, 2491-2497.

41. Ciesielski, G.L., Bermek, O., Rosado-Ruiz, F.A., Hovde, S.L., Neitzke, O.J., Griffith, J.D. and Kaguni, L.S. (2015) Mitochondrial Single-stranded DNA-binding Proteins Stimulate the Activity of DNA Polymerase gamma by Organization of the Template DNA. *J Biol Chem*, **290**, 28697-28707.

42. Baqri, R.M., Turner, B.A., Rheuben, M.B., Hammond, B.D., Kaguni, L.S. and Miller, K.E. (2009) Disruption of mitochondrial DNA replication in Drosophila increases mitochondrial fast axonal transport in vivo. *PLoS One*, **4**, e7874.

43. Bratic, A., Kauppila, T.E., Macao, B., Gronke, S., Siibak, T., Stewart, J.B., Baggio, F., Dols, J., Partridge, L., Falkenberg, M. *et al.* (2015) Complementation between polymerase- and exonuclease-deficient mitochondrial DNA polymerase mutants in genomically engineered flies. *Nat Commun*, **6**, 8808.

44. Siibak, T., Clemente, P., Bratic , A., Bruhn, H., Kauppila, T.E.S., Macao, B., Schober, F.A., Lesko, N., Wibom, R., Naess, K. *et al.* (2017) A multi-systemic mitochondrial disorder due to a dominant p.Y955H disease variant in DNA polymerase gamma. *Hum Mol Genet.*, **26**, 2515-2525.

45. Wang, Y., Farr, C.L. and Kaguni, L.S. (1997) Accessory subunit of mitochondrial DNA polymerase from Drosophila embryos. Cloning, molecular analysis, and association in the native enzyme. *J Biol Chem*, **272**, 13640-13646.

46. Lee, Y.S., Lee, S., Demeler, B., Molineux, I.J., Johnson, K.A. and Yin, Y.W. (2010) Each monomer of the dimeric accessory protein for human mitochondrial DNA polymerase has a distinct role in conferring processivity. *J BIol Chem.*, **285**, 1490-1499.
